# A Web-Based Platform for Designing Vaccines against Existing and Emerging Strains of *Mycobacterium tuberculosis*

**DOI:** 10.1371/journal.pone.0153771

**Published:** 2016-04-20

**Authors:** Sandeep Kumar Dhanda, Pooja Vir, Deepak Singla, Sudheer Gupta, Shailesh Kumar, Gajendra P. S. Raghava

**Affiliations:** Bioinformatics Centre, Institute of Microbial Technology, Chandigarh, 160036, India; Colorado State University, UNITED STATES

## Abstract

Development of an effective vaccine against drug-resistant *Mycobacterium tuberculosis* (Mtb) is crucial for saving millions of premature deaths every year due to tuberculosis. This paper describes a web portal developed for assisting researchers in designing vaccines against emerging Mtb strains using traditional and modern approaches. Firstly, we annotated 59 genomes of Mycobacterium species to understand similarity/dissimilarity between tuberculoid, non-tuberculoid and vaccine strains at genome level. Secondly, antigen-based vaccine candidates have been predicted in each Mtb strain. Thirdly, epitopes-based vaccine candidates were predicted/discovered in above antigen-based vaccine candidates that can stimulate all arms of immune system. Finally, a database of predicted vaccine candidates at epitopes as well at antigen level has been developed for above strains. In order to design vaccine against a newly sequenced genome of Mtb strain, server integrates three modules for identification of strain-, antigen-, epitope-specific vaccine candidates. We observed that 103522 unique peptides (9mers) had the potential to induce an antibody response and/or promiscuous binder to MHC alleles and/or have the capability to stimulate T lymphocytes. In summary, this web-portal will be useful for researchers working on designing vaccines against Mtb including drug-resistant strains. Availability: The database is available freely at http://crdd.osdd.net/raghava/mtbveb/.

## Introduction

The tuberculosis cost millions of lives every year globally as well as billions of humans are infected with this deadly disease [1,2]. In addition, treatment is not patient friendly, as it requires combination of drugs for six months. The emergence of drug-resistant Mtb strains further complicating the problem as existing drugs are not effective against these strains [3,4]. Fortunately, BCG based vaccination against tuberculosis is available since 1921 that is responsible for saving millions of death every year [5]. BCG based vaccine is highly protective in case of children and in developed countries, but the efficacy is questionable for adults, immunocompromised individuals, and the residents of developing nations where it was most needed [6]. There are various reasons suggested for the BCG failures, some of them are as variation in the strains of BCG vaccine (nobody knows which strains is best protective), environmental exposure to Nontuberculous Mycobacteria (NTM) strains (interfere with immune response to be elicited by vaccine strain possibly due to neutralizing antibodies) [7,8]. There is a need to address this problem in modern era using latest techniques like next generation sequencing (NGS) and virtual screening of epitopes.

In the era NGS, the entire microbial genomes can be sequenced at affordable cost within hours [9]. These newly sequenced strains may have the potential to act as the vaccine strain, but there is no platform for predicting vaccine potential of a strain. In the literature, some key points have been mentioned as the requirement of a vaccine strain for tuberculosis [10]. These points include the involvement of virulence factors, ESX system [11] and the proteins from region of genetic variance [12]. The second challenge for tuberculosis vaccine is desired immune response. The desired immune response to tuberculosis vaccine has been reported in the literature [13–15]. The desired immunity includes cell-mediated immune response characterized by MHC binders and Th1 activating environment [16–18]. The role humoral immunity is also not clearly understood and well established [6,19].

In this study, we have made an attempt to develop a comprehensive database or web portal for designing vaccine against large number of mycobacterium strains whose whole genome have been already sequenced. In addition, tools have been integrated in this web portal that allow users to identify vaccine candidates against a new strain (e.g., emerging strain, drug resistant) from their whole genome sequence. In this study, first we identified 178 antigens based on previous studies, which are potential vaccine candidates. We developed immune-informatics pipeline that automatically predict different types of antigenic regions or epitopes required for activating different arms of immune system. Identification of conserved regions in a vaccine candidate is one of the challenge in designing vaccine against a strain of *Mycobacterium*[20]. In order to address this issue our server aligns new antigen/vaccine candidates against similar antigens in other strains of *Mycobacterium* for identification of conserved region from multiple sequence alignment. In summary our database provides vaccine related information about 59 strains and provide facility to identify vaccine against new strain from their genome sequence. Therefore, the aim of the present study is to develop a web portal for comprehensive study of mycobacterial strains and vaccine candidates for designing better vaccine against tuberculosis.

## Methods

### Strains

We downloaded whole genome of 59 Mtb strains with complete (chromosome) annotation from the NCBI [21]. We selected genomes based on certain criteria’s that includes availability of complete genome in NCBI and annotation of genome at chromosome level. These strains belong to diverse category of pathogenesis, drug-resistance, drug-sensitive, clinical isolates, lab strains, non-tuberculoid and vaccine strains (Figure A in S1 File). Following is brief description of these three types strains

#### Tuberculoid strain

The mycobacterium strains that can cause tuberculosis infection are called tuberculoid strains. We compiled wide-range of tuberculoid strains that includes drug-sensitive, multi drug-resistant, extensively drug-resistant and clinical isolates. In this study we have collected and compiled genomics information about 23 tuberculoid strains.

#### Vaccine strains

In this study, we compiled genomic information about five M. bovis vaccine strains (e.g., AF2122/97, BCG strain Korea, BCG strain Tokyo 172). These tuberculosis vaccine strains are attenuated bovine strains named as Bacillus Calmette Guerin (BCG). The vaccine strain is sub-cultured in many labs around the world and had undergone several variations in the genome. Among them, four vaccine variants have been sequences and annotated at chromosome level. We have categories these four variants and their parental vaccine strains under vaccine strain groups.

#### Non-tuberculoid strains

The mycobacterial strains that do not cause active tuberculosis in human are called non-tuberculoid mycobacterium (NTM). Most of the NTM are environmental mycobacteria, but some of NTM may be opportunistic pathogen, which can cause disease in immune compromised individuals. Here, we have total 30 NTM strains that include the strains causing leprosy and ulcer.

### Vaccine candidates

One of the challenges in designing subunit-vaccine is identification of potential vaccine candidate. Though a mycobacterium strain have around 4000 proteins only limited proteins are potential vaccine candidates [22]. We collected and compiled potential vaccine candidates from previous studies [14]. In this study, we have taken strain H37Rv as reference strain for identification of potential vaccine candidates. These vaccine candidates belong to three categories as given below.

#### Virulence associated proteins

The proteins associated with the virulence of tuberculosis are attractive vaccine targets. PPE family serves as the best example, which has been explored widely for vaccine potential [23–25]. We have obtained the 125 virulence factors from Mycobacterium tuberculosis which were beautifully summarized in review article [26].

#### Components of secretion system

Secretion system from tuberculosis is extensively exploited for hunting a new vaccine candidate and as a result some of the candidates like ESAT-6 and antigen85 are running in clinical trials [27–29]. Champion and Cox had reviewed the protein secretion system from *Mycobacterium tuberculosis* [30]. We have extracted the 20 components of secretion system from this review for vaccine candidates.

#### Regions of deletion

The BCG vaccine has been developed from a bovine virulent strain. During the process of attenuation several proteins has undergone mutation and variation. The region of deletion (RD) is supplemented to BCG to create recombinant BCG (rBCG) to provide better protection [31–33]. Gunawardena *et*. *al*. compared the membrane proteins of virulent and vaccine strains of tuberculosis [34]. They identified proteins that show genetic variance between virulent and vaccine strains. In this study, we obtained 33 vaccine targets from above study.

Overall we obtained total 178 (166 unique) vaccine candidates for Mycobacterium strain H37Rv, which contain 125 virulent, 22 secretory and 33 RD proteins.

#### Vaccine candidates in other strains

In order to identify vaccine candidates in other strains of Mycobacterium, we perform similarity search of these 178 vaccine candidates (in strain H37Rv) against whole proteome of these strains. Similarity, search was performed using needleall software of emboss package and selected those proteins as vaccine candidate that have highest identity with cut-off 95%. The numbers of vaccine candidates identified using above similarity search is shown in Table A in S1 File.

### Epitope or antigenic regions

Experimentally validated epitopes: We obtained experimentally validated or characterize epitopes from Immune Epitope Database (IEDB), largest database of epitopes [35]. These epitopes were collected from four types of assays includes B-cell response, MHC binding and T cell response assays. We map these epitopes on vaccine candidates to identify antigenic regions or epitopes in vaccine targets.

#### Prediction of epitopes

In order to predict epitopes in vaccine targets, first we generate all possible overlapping 9mer peptides in vaccine targets. Next, we remove redundant or duplicate peptides from these 9mer peptides. Finally, we used a pipeline for predicting different types of epitopes in these 9mer peptides. This pipeline predict; i) B-cell epitopes using Lbtope [36], ii) MHC class I & II binders using Propred/Propred1 [37–39] and iii) T-cell epitopes using software CTLpred, IFNepitope and IL4pred [40–42]. We have selected the peptides with desired immune response; see Figure B in S1 File for flow diagram used in this study.

### Database architecture & platform

**Fig 1:**
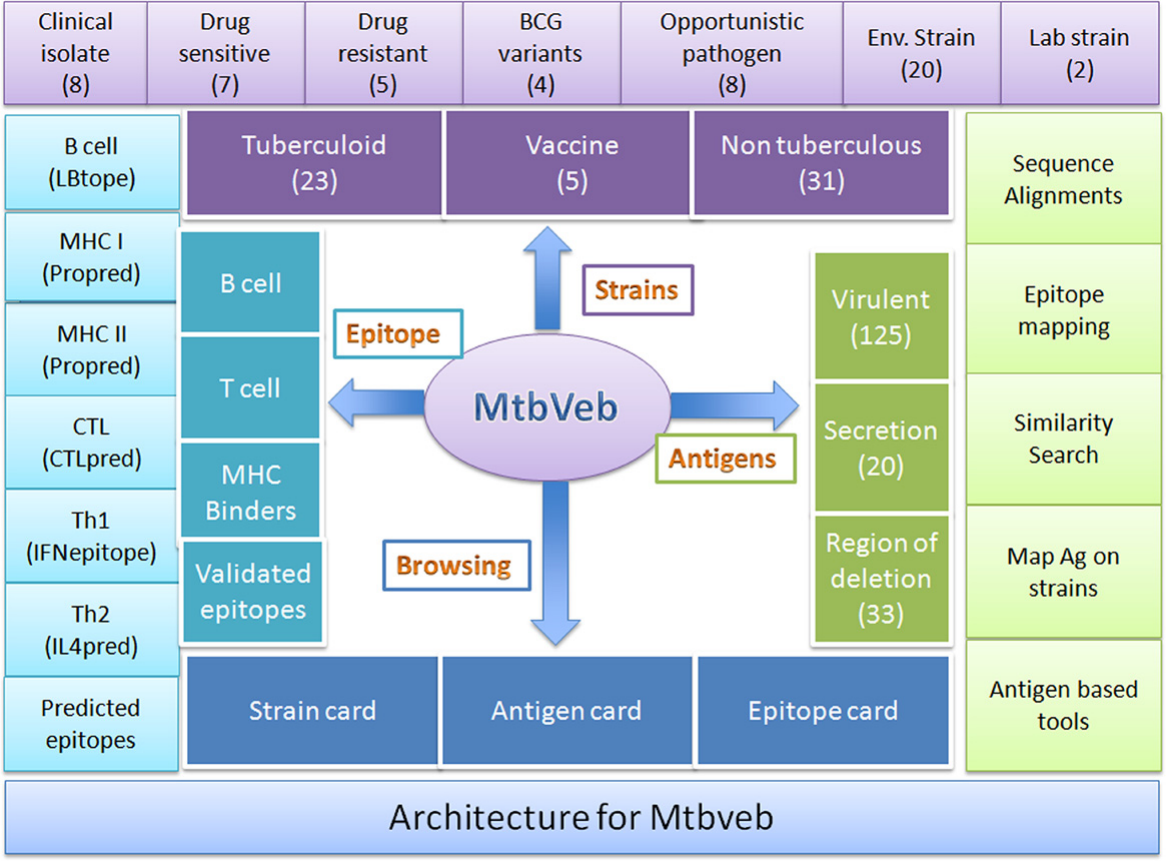
Architecture of database for MtbVeb platform.

The architecture of database is shown in Fig 1 that organized data in three categories strain, vaccine candidates and epitopes. In order to manage data efficiently, we used relational data management system (RDBM) implemented using MySQL server. This web site is launched using Apache HTTP Server 2.2 on a linux operating system. All web interfaces has been developed using HTML and PHP 5.2.9. In addition JavaScript is used for number of functions including visualizing of genomes.

## Result

### Analysis of vaccine candidates

**Fig 2:**
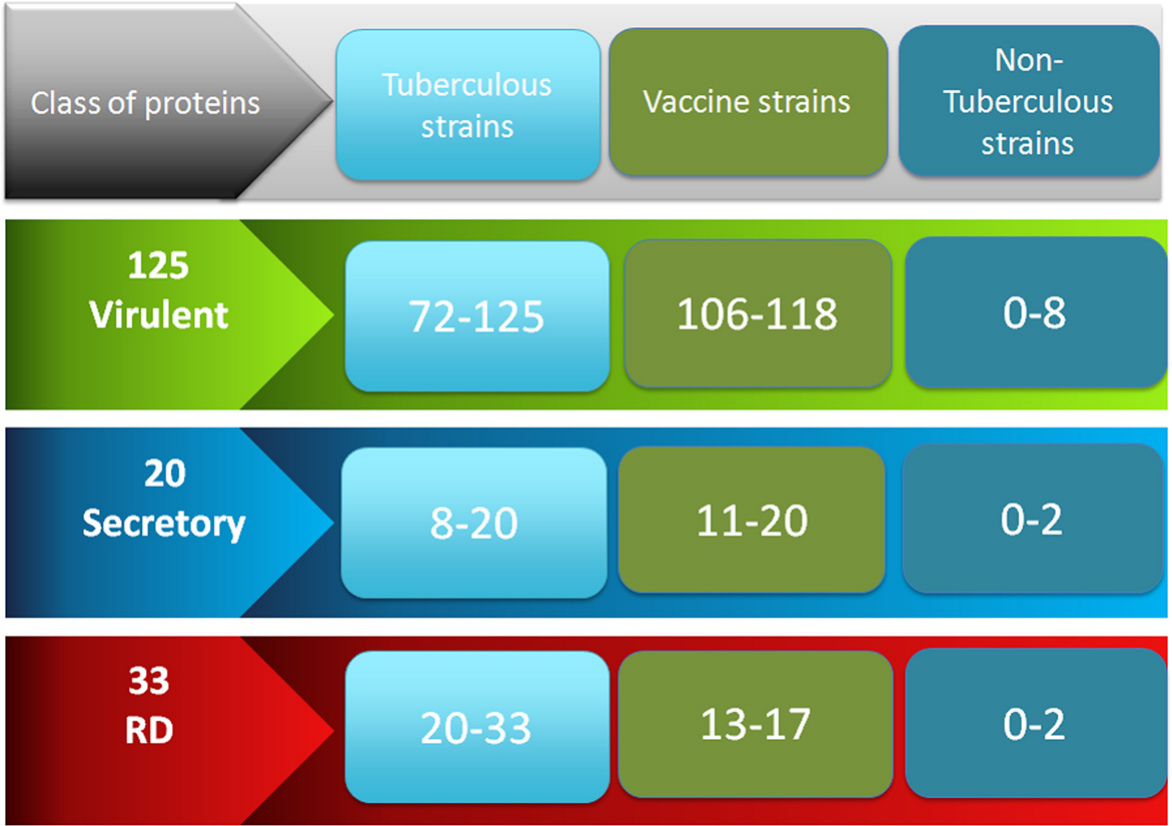
Distribution of virulent proteins, secretion system proteins and proteins of genetic variance among three category of strains.

We compute length-wise distribution of 166 unique vaccine targets (Total 178) to understand length-wise distribution. It was observed that length of vaccine candidates varies from 48 to 2126 amino acids. Most of the vaccine candidates have number residues between 100 to 600 (Figure C in S1 File). We examine whether these three types of vaccine targets (125 virulent, 20 secretory and 33 RD proteins) can be used to discriminate different types of strains. Thus we predict presence of these antigens in each strain based on their similarity with target antigens in tuberculosis strain. It was observed that 125 virulent and 20 secretory proteins can discriminate pathogenic and non-pathogenic variants of mycobacteria, but these proteins could not differentiate vaccine strains from the pathogenic strains (Fig 2 and Figure B and Table C in S1 File). Our 33 vaccine targets RD proteins could discriminate vaccine strains from the pathogenic strains (Table D in S1 File). The supplementary data shows the number of homologous proteins (with sequence identity cut off 95 percent) extracted out of various pathogenic strain and these homologs were then searched in different non-tuberculoid mycobacterial (NTM) strains. It is to be noted here that there were no difference in number of proteins in *Mycobacterium africanum GM041182* (tuberculoid strain) [43] and variant of BCG, when we searched 125 virulent proteins and 20 ESX secretion system proteins. On the other hand, we observed significant difference when we searched the homologs of 33 proteins of genetic variance.

We annotate virulent proteins to understand their function and role in bacterial system, information was extracted from UniProt database [44]. We extracted detailed about each protein like cellular components, pathways, motifs, link to other databases, and gene ontology (Figure D in S1 File). We end up 125 proteins, which localized in various part of the cell as depicted in Figure E in S1 File. As it evident from Fig 2 that half of our proteins are component of plasma membrane or cell wall and 11% are extracellular proteins.

### Epitope mapping and prediction

We mapped experimentally validated epitopes (available in IEDB database) on 59 strains of mycobacterium strains (Table 1, Figure F in S1 File). As shown in Table 1, 659 B-cell epitopes in IEDB mapped on 30992 segments of mycobacterium strains. It is interesting to note that out of 659 epitopes, 628 epitopes were mapped on vaccine candidates. It means most of B-cell epitopes were obtained from candidates. In case of T-cell epitopes, 1806 were mapped on strains and 1063 on vaccine candidates. In addition to mapping of experimentally validated epitopes, we also predicted epitopes in above vaccine candidates using prediction software. First, we generate unique 9mer peptides in vaccine candidates in all strains and obtained total 103522 unique peptides (Table 2). Out of these peptides, 8292 peptides were predicted to be linear B-cell epitopes. There were 14907 potential MHC class II binders, 46484 MHC class I binders. Out of 46484 MHC class I binders, 34922 peptides were predicted to induce cytotoxic T lymphocytes. On the other hand, there were 391 peptides that can produce Th1 immunity and humoral response. We hope these peptides have the potential for a subunit vaccine.

**Table 1:** Mapping of experimentally characterized epitopes/MHC-binders on all antigens and vaccine candidates of mycobacterium strains.

Epitopes / Binders	Whole Mycobacterium Strains		Only on Vaccine Candidates
	Total Peptides	Unique Peptides	Unique Peptides
**B-cell epitope**	30992	659	628
**T-cell epitope**	142265	1806	1063
**MHC binders**	180726	1208	387

**Table 2:** List of 9mer peptides, epitopes, MHC binders in vaccine candidates.

Filter ID	Filter	Genetic Variance	Secretion	Virulent	Total (Unique)
ID1	Proteins	33	20	125	166
ID2	9mer peptides	230509	121895	1366365	1718769
ID3	Unique 9mer	12941	7566	87097	103522
ID4	LBtope	1090	661	6899	8292
ID5	Propred1	8954	5029	34899	46484
ID6	CTLpred	2132	1000	55839	58492
ID7 (ID5 + ID6)	CD8 epitopes	2028	969	32329	34922
ID8	Propred	1847	968	12632	14907
ID9	IFNepitope	5276	3114	36594	43489
ID10 (ID8 + ID9)	Th1 epitopes	776	359	5294	6242
ID11	IL4pred	8769	4852	57089	67952
ID12 (ID8 + ID11)	Th2 epitopes	1241	619	8216	9720
**ID13 (ID10 + ID4)**	**Th1 + B cell**	**62**	**25**	**318**	**391**
**ID14 (ID10—ID4)**	**Th1—B cell**	**714**	**334**	**4976**	**5851**

### Web Implementation of MtbVeb

In this database, data is compiled in three sections for designing strain, antigen and epitope based vaccine. In order to retrieve and analysis vaccine related data in our database, we integrated various web-based tools. Following is the brief description of menus and submenus incorporated in MtbVeb database for designing vaccines based on strain, antigen and epitope.

### Strain-specific information

This menu allows users to identify most appropriate strain for designing vaccine against a pathogenic strain. It also allows users to analysis their newly sequenced mycobacterium strain. This menu comprise various submenus allow users to access data at strain level. First submenu “Browse on strains” list all strains maintained in this database with their type and brief description. User can check experimentally validated epitopes mapped on different strains using “Browse on Epitopes” submenu. In order to compare vaccine targets in different strains, server presents multiple sequence alignment of each target in different strains. This can be achieved using “Strain-wise comparison” submenu; in addition user can identify conserved regions in vaccine targets from their multiple sequence alignment. Submenus identify vaccine strain, designed for identification of appropriate vaccine strain based on different class of vaccine candidates present in these strains. Analysis of user strain is an important submenu that allows users to analysis genomic information provides in any form like short reads, contigs. We integrated three well-known genome browsers (e.g., JBrowse, CGView, Argo) in our database for visualization of genome of strains.

### Antigen-based vaccines

In order to develop antigen-based vaccine against a pathogenic strain, we integrate tools for identification of most appropriate vaccine candidates. This menu has number of submenus to retrieve comprehensive information about each antigen in any mycobacterium strains. Once can retrieve sequence related information of 178 vaccine candidates or antigens in a strain using submenu “Browse on Antigens”. User may get detail information about each antigen using antigen card. Submenu “Browse Vaccine Targets” of server shows presence or absence of vaccine targets in 59 mycobacterium strains. Similarly, other submenus under this menu allow users to perform similarity search, alignment and epitope mapping on query sequence. [45].

### Epitope Prediction

In present era major focus of researchers in on micro minimization of vaccines from strain to antigen to epitope. This menu developed for predicting epitopes suitable for designing epitope-based vaccine against a mycobacterium strain. The browse submenu allow users map predict epitopes on vaccine candidates; it includes predicted B-cell and T-cell epitopes. It is possible to perform complex search on predicted epitopes using submenu “Advance Search”. We allow users to list most potential epitopes having potential to activate desired immune response. User can predict epitopes in their antigen sequence using prediction pipeline used in this study.

### Limitation of MtbVeb

Although we have tried our best to develop a platform to provide comprehensive information required for developing vaccine or immunotherapy against *M*. *tuberculosis*. Still it has number of limitations that need to be addressed in future studies. This platform provides no information about the formulation of peptides with different adjuvants for better immunization. Due to technological advances whole genome sequencing of strains is growing with exponential rate. Thus server needs to be updated frequently to incorporate information about newly sequenced strains.

## Discussion

Presently only limited options are available to fight against deadly infectious disease tuberculosis; BCG is only vaccine. In past it has been shown that BCG provides limited protection against tuberculosis particularly adults. There is a continuous effort by scientific community to improve BGC strains in order to enhance effectiveness [46,47]. Alternatively, researchers are searching new vaccine strains, which may provide protection against pathogenic strains particularly against emerging drug resistant strains. Fortunately whole genome of wide range of mycobacterium strain has been sequenced and available in public domain. There is a need to analysis whole genome sequence of theses newly sequenced strain for identification of most appropriate vaccine strain. In this study we made a systematic attempt to develop computational tools and database for identification vaccine strain against a given pathogenic strain.

In addition to strain-specific vaccines, researchers are focusing on antigen-based vaccines where major emphasis is on identification of potential antigen. Several vaccine candidates have been identified in previous studies and some of these candidates are also running in clinical trials [48–52]. In the past, researchers have developed databases and prediction tools pertaining to tuberculosis vaccine research. He et al. developed a platform named as ‘Violin’ for vaccine information [53]. Violin covers several aspects of vaccines like vaccine components, mechanism, design, and community. For tuberculosis vaccine policies, Zwerling et al. has developed a tool [54]. MycobacRv is another database related to TB vaccine [55]. MycobacRv has limits its focus to adhesion proteins in the genomes of different mycobacterial species. None of the existing tools is providing a solution to design a vaccine for emerging strains. As shown in Table 3, our MtbVeb had more features than existing resources. Thus our server will complement existing resources in the field of vaccines against *M*. *tuberculosis*. The major facilities in our web platform includes, i) prediction of new epitopes, ii) mapping of already known epitope and iii) automatic pipeline for identification of epitopes. One of the major features of our web server is analysis of data at antigen and strain-level.

**Table 3:** Comparison of different databases available for tuberculosis vaccination.

Feature	Violin	Atlas	MycobacRV	MtbVeb
BCG policies	No	Yes	No	No
Vaccine candidates	Yes	No	Yes	Yes
Strains-specific analysis	Yes	No	Yes	Yes
Comparison with BCG	No	No	No	Yes
Prediction of epitopes	No	No	Yes	Yes
User-based strain analysis	No	No	No	Yes
Mapping of experimental epitopes	No	No	No	Yes

In this study we also made systematic attempt to design antigen and epitope based vaccine. First we identified potential vaccine candidates from literature that have been extensively studied in past. These vaccine candidates have been searched in all strains whose genome sequence is available. All the information required for developing antigen-based vaccines against mycobacterium strains have been compiled in for of a database MtbVeb. We also mapped experimentally validated and predicted epitopes on above potential vaccine candidates. This information will be very useful in designing epitope-based vaccine against any emerging or existing strain of mycobacterium. We have also filtered 12 peptides that have shown diverse immunity and present in number of tuberculoid strain, but absent in vaccine strains (Table E in S1 File). In summary our database cum web-based platform provides comprehensive information on 59 mycobacterium strains. This comprehensive information and tools integrated in MtbVeb allow one to design computer-aided vaccine based on whole strain, antigen and epitope.

## Supporting Information

S1 File Supplementary file containing Figures A-F and Tables A-E. (DOCX)
