# Supplementary material for: A Web-Based Platform for Designing Vaccines against Existing and Emerging Strains of Mycobacterium tuberculosis
Source: PLoS One. 2016 Apr 20;11(4):e0153771. doi: 10.1371/journal.pone.0153771 (PMC4838326; doi:10.1371/journal.pone.0153771)
Supplement: S1 File — (DOCX) [file pone.0153771.s001.docx]

**A web-based platform for designing vaccines against existing and emerging strains of *Mycobacterium tuberculosis***

Sandeep Kumar Dhanda, Pooja Vir, Deepak Singla, Sudheer Gupta, Shailesh Kumar and Gajendra P.S. Raghava*

* **Address for correspondence**

Gajendra P.S. Raghava

Scientist and Head, Bioinformatics Centre,

CSIR-Institute of Microbial Technology,

Sector 39A, Chandigarh, India

Email: [raghava@imtech.res.in](mailto:raghava@imtech.res.in)

Web: <http://www.imtech.res.in/raghava/>

Phone: +91-172-2690557

Fax: +91-172-2690632

# Supplementary Information

## List of Figures

Figure A: Pie Chart to display distribution of strain among different category of virulence

Figure B: Flow diagram for selection of peptide-based subunit vaccine candidates

Figure C: Histogram plot for the distribution of vaccine candidates from Mtb H37Rv according to their residue length.

Figure D: Depicting the pathways-wise distribution of vaccine candidates. The number has been generated based on the data from UniProt and for the (21) proteins to which pathways have been assigned.

Figure E: Representing the localization of virulent proteins in a cell. Some of the proteins have been shown to present two or more location in the cell, and they are on all the location, where they have been mentioned in UniProt.

Figure F: Distribution of IEDB mapped B cell, T cell and MHC binding assays as well as epitopes in any of the Mycobacterial strains

## List of Tables

Table A: The vaccine targets from all strains of tuberculosis at 95% sequence identity searched using EMBOSS needleall algorithm.

Table B: Searching the number of virulent proteins of each tuberculoid strain from Non tuberculoid mycobacteria and vaccine strains. We have included *M africanum* GM041182 virulent strain as a representative of virulent strains.

Table C: Searching the number of secretory proteins of each tuberculoid strain from Non tuberculoid mycobacteria and vaccine strains. We have included *M africanum* GM041182 virulent strain as a representative of virulent strains

Table D: Searching the number of regions of variance of each tuberculoid strain from Non tuberculoid mycobacteria and vaccine strains. We have included *M africanum* GM041182 virulent strain as a representative of virulent strains.

Table E: Selected vaccine candidate with their predicted immune response


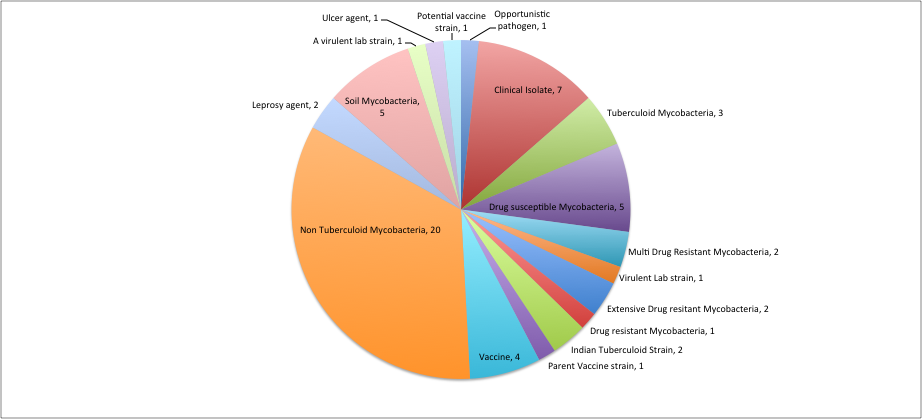


Figure A: Pie Chart to display distribution of strain among different category of virulence


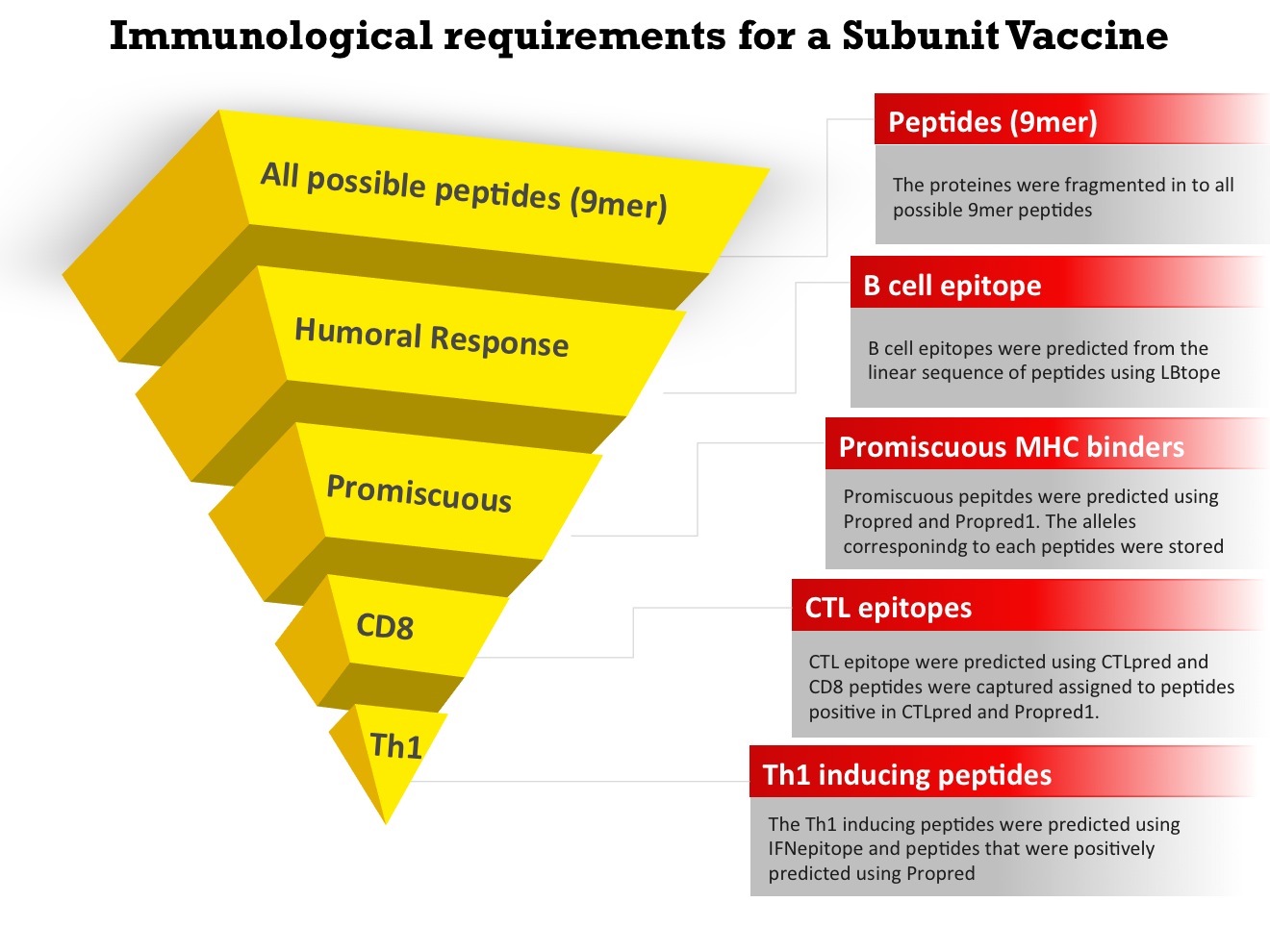


Figure B: Flow diagram for selection of peptide-based subunit vaccine candidates

###
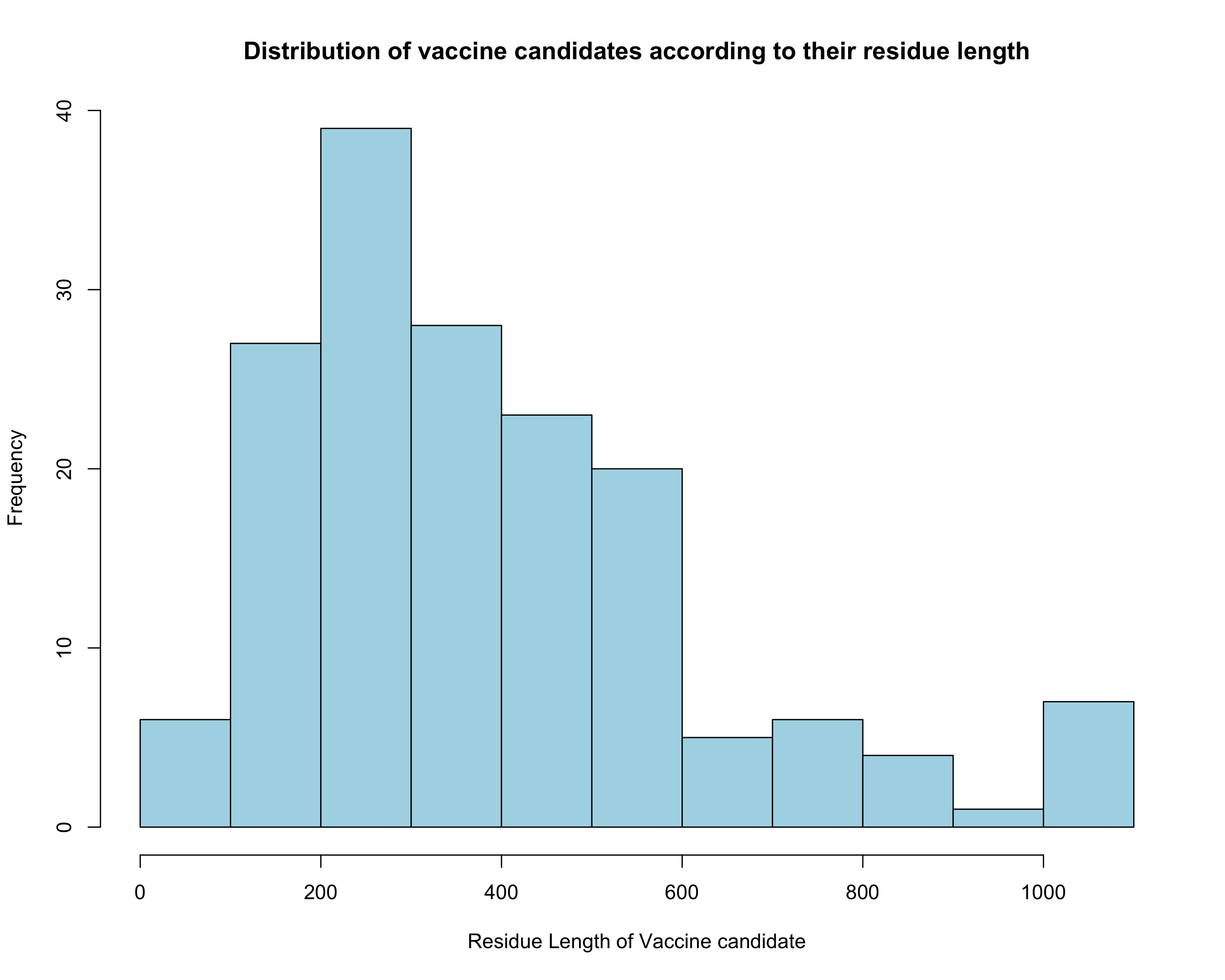


Figure C: Histogram plot for the distribution of vaccine candidates from Mtb H37Rv according to their residue length.


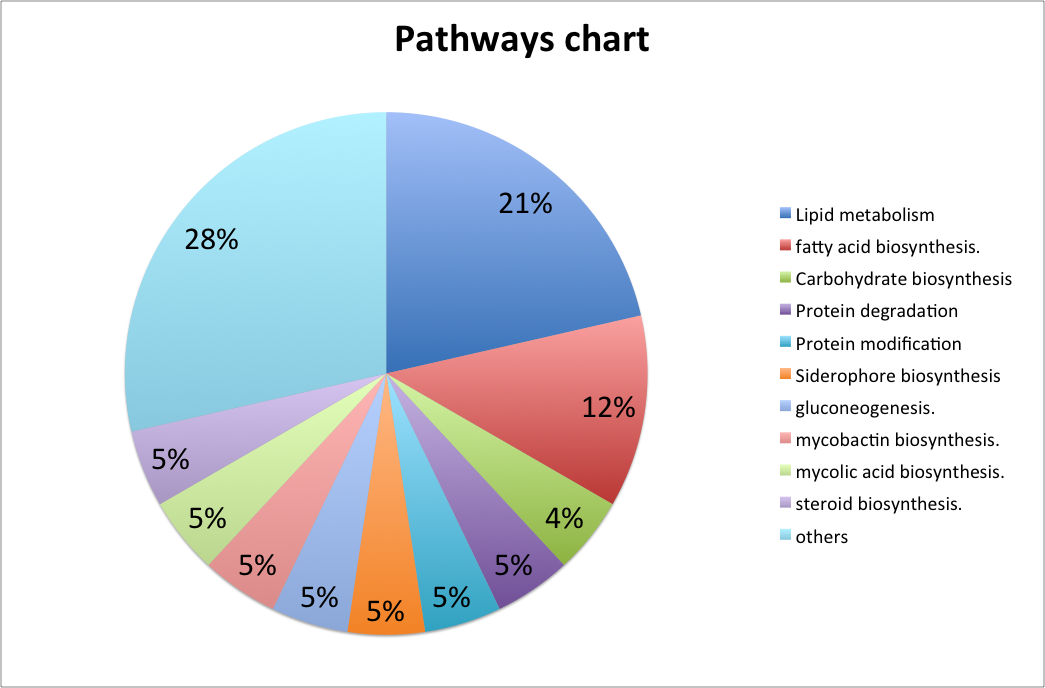


Figure D: Depicting the pathways-wise distribution of vaccine candidates. The number has been generated based on the data from UniProt and for the (21) proteins to which pathways have been assigned.


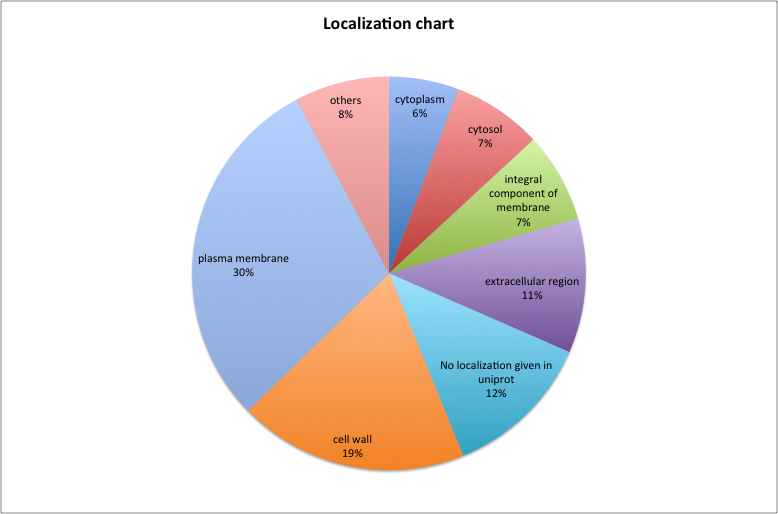


Figure E: Representing the localization of virulent proteins in a cell. Some of the proteins have been shown to present two or more location in the cell, and they are on all the location, where they have been mentioned in UniProt.

**
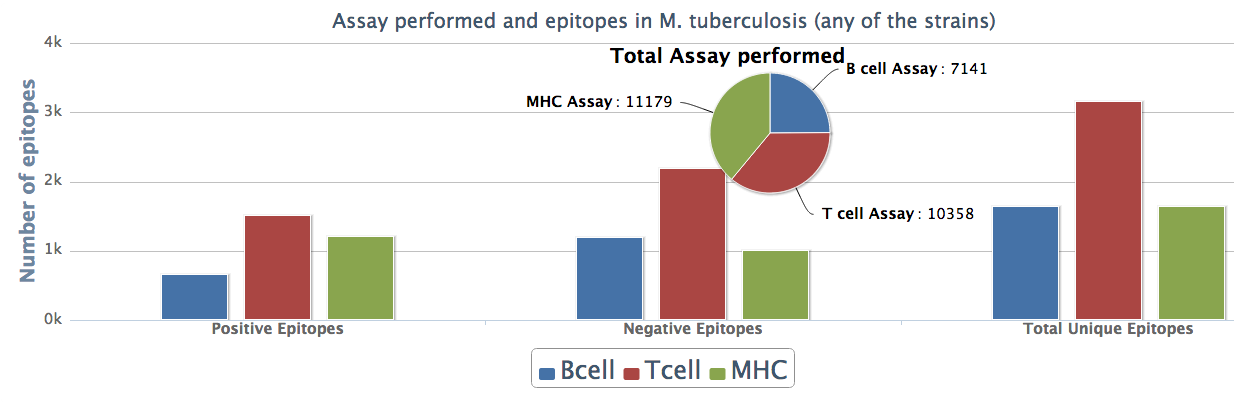
**

Figure F: Distribution of IEDB mapped B cell, T cell and MHC binding assays as well as epitopes in any of the Mycobacterial strains

Table A: The vaccine targets from all strains of tuberculosis at 95% sequence identity searched using EMBOSS needleall algorithm.

| **Strain** | **No. of virulent proteins** | **No. of secretory proteins** | **No. of regions from genetic variance** |
| --- | --- | --- | --- |
| M_abscessus | 1 | 0 | 0 |
| M_africanum_GM041182 | 107 | 10 | 23 |
| M_avium_104 | 3 | 0 | 0 |
| M_avium_K-10 | 3 | 0 | 0 |
| M_bovis_AF2122_97 | 118 | 20 | 17 |
| M_bovis_BCG_str_Korea | 106 | 11 | 13 |
| M_bovis_BCG_str_Mexico | 112 | 12 | 14 |
| M_bovis_BCG_str_Pasteur_1173P2 | 113 | 12 | 14 |
| M_bovis_BCG_str_Tokyo_172 | 114 | 12 | 14 |
| M_canettii_CIPT_140010059 | 103 | 10 | 25 |
| M_canettii_CIPT_140010059_3861 | 110 | 10 | 29 |
| M_canettii_CIPT_140060008 | 84 | 8 | 20 |
| M_canettii_CIPT_140070008 | 100 | 10 | 25 |
| M_canettii_CIPT_140070010 | 101 | 10 | 24 |
| M_canettii_CIPT_140070017 | 113 | 19 | 27 |
| M_chubuense_NBB4 | 1 | 0 | 0 |
| M_gilvum_PYR-GCK | 0 | 0 | 0 |
| M_gilvum_Spyr1 | 0 | 0 | 0 |
| M_indicus_prani_MTCC_9506 | 2 | 0 | 1 |
| M_intracellulare_ATCC_13950 | 2 | 0 | 1 |
| M_intracellulare_MOTT-02 | 3 | 0 | 1 |
| M_intracellulare_MOTT-64 | 2 | 0 | 1 |
| M_kansasii_ATCC_12478 | 8 | 2 | 0 |
| M_leprae_Br4923 | 1 | 0 | 0 |
| M_leprae_TN | 1 | 0 | 0 |
| M_liflandiii_128FXT | 3 | 1 | 1 |
| M_marinum | 3 | 1 | 2 |
| M_massiliense_GO_06 | 0 | 0 | 0 |
| M_rhodesiae_NBB3 | 0 | 0 | 0 |
| M_smegmatis_JS623 | 0 | 0 | 0 |
| M_smegmatis_MC2_155 | 0 | 0 | 0 |
| M_smegmatis_MKD8 | 0 | 0 | 0 |
| M_smegmatis_str_MC2_155 | 0 | 0 | 0 |
| M_smegmatis_str_MC2_155_second | 0 | 0 | 0 |
| M_ulcerans_Agy99 | 2 | 0 | 1 |
| M_vanbaalenii_PYR-1 | 0 | 0 | 0 |
| Mtb_7199-99 | 125 | 20 | 33 |
| Mtb_CCDC5079 | 99 | 15 | 23 |
| Mtb_CCDC5180 | 105 | 16 | 26 |
| Mtb_CDC_1551 | 110 | 14 | 29 |
| Mtb_CTRI-2 | 121 | 20 | 30 |
| Mtb_F11 | 116 | 19 | 29 |
| Mtb_H37Ra | 122 | 20 | 33 |
| Mtb_H37Rv | 125 | 20 | 33 |
| Mtb_H37Rv_broad | 114 | 11 | 30 |
| Mtb_KZN_1435 | 116 | 20 | 29 |
| Mtb_KZN_4207 | 109 | 10 | 28 |
| Mtb_KZN_4207-3996 | 117 | 20 | 30 |
| Mtb_KZN_605 | 119 | 20 | 30 |
| Mtb_KZN_R506 | 118 | 17 | 29 |
| Mtb_KZN_V2475 | 118 | 17 | 30 |
| Mtb_RGTB327 | 77 | 13 | 22 |
| Mtb_RGTB423 | 72 | 13 | 24 |
| Mtb_UT205 | 120 | 20 | 31 |
| Mycobacterium_sp_JDM601 | 0 | 0 | 0 |
| Mycobacterium_sp_JLS | 0 | 0 | 0 |
| Mycobacterium_sp_KMS | 0 | 0 | 0 |
| Mycobacterium_sp_MCS | 0 | 0 | 0 |
| Mycobacterium_sp_MOTT36Y | 2 | 0 | 1 |
| Total | 3321 | 453 | 773 |

Table A: Searching the number of virulent proteins of each tuberculoid strain from Non tuberculoid mycobacteria and vaccine strains. We have included *M africanum* GM041182 virulent strain as a representative of virulent strains.

|  |
| --- |

| **Tb strain** | **No. of virulent proteins** | **M_africanum_GM041182** | **M bovis AF2122_97** | **M bovis BCG Korea** | **M bovis BCG Mexico** | **M bovis BCG Pasteur_1173P2** | **M bovis BCG Tokyo_172** |
| --- | --- | --- | --- | --- | --- | --- | --- |
| M_africanum_GM041182 | 107 | 107 | 103 | 92 | 100 | 100 | 101 |
| M_canettii_CIPT_140010059_3861 | 110 | 98 | 102 | 92 | 98 | 99 | 100 |
| M_canettii_CIPT_140010059 | 103 | 90 | 96 | 85 | 91 | 92 | 93 |
| M_canettii_CIPT_140060008 | 84 | 71 | 81 | 73 | 77 | 78 | 79 |
| M_canettii_CIPT_140070008 | 100 | 93 | 94 | 85 | 91 | 91 | 92 |
| M_canettii_CIPT_140070010 | 101 | 85 | 94 | 86 | 90 | 90 | 91 |
| M_canettii_CIPT_140070017 | 113 | 89 | 95 | 84 | 92 | 93 | 94 |
| Mtb_7199-99 | 125 | 106 | 117 | 105 | 111 | 112 | 113 |
| Mtb_CCDC5079 | 99 | 83 | 93 | 88 | 89 | 89 | 90 |
| Mtb_CCDC5180 | 105 | 86 | 95 | 91 | 92 | 92 | 93 |
| Mtb_CDC_1551 | 110 | 95 | 102 | 92 | 98 | 98 | 99 |
| Mtb_CTRI-2 | 121 | 104 | 115 | 101 | 109 | 110 | 111 |
| Mtb_F11 | 116 | 100 | 109 | 101 | 105 | 105 | 106 |
| Mtb_H37Rv | 125 | 106 | 117 | 105 | 111 | 112 | 113 |
| Mtb_H37Rv_broad | 114 | 99 | 106 | 97 | 102 | 102 | 103 |
| Mtb_KZN_1435 | 116 | 98 | 108 | 101 | 103 | 103 | 104 |
| Mtb_KZN_4207-3996 | 117 | 100 | 110 | 101 | 105 | 105 | 106 |
| Mtb_KZN_4207 | 109 | 97 | 102 | 95 | 99 | 99 | 100 |
| Mtb_KZN_605 | 119 | 102 | 112 | 103 | 107 | 107 | 108 |
| Mtb_KZN_R506 | 118 | 103 | 112 | 101 | 108 | 108 | 109 |
| Mtb_KZN_V2475 | 118 | 102 | 110 | 101 | 106 | 106 | 107 |
| Mtb_RGTB327 | 77 | 67 | 73 | 66 | 72 | 72 | 72 |
| Mtb_RGTB423 | 72 | 64 | 68 | 58 | 64 | 64 | 65 |
| Mtb_UT205 | 120 | 102 | 113 | 99 | 107 | 108 | 109 |

Table C: Searching the number of secretory proteins of each tuberculoid strain from Non tuberculoid mycobacteria and vaccine strains. We have included *M africanum* GM041182 virulent strain as a representative of virulent strains

| **Tb strain** | **No. of secretion proteins** | **M_africanum_GM041182** | **M_bovis_AF2122_97** | **M_bovis_BCG_str_Korea** | **M_bovis_BCG_str_Mexico** | **M_bovis_BCG_str_Pasteur_1173P2** | **M_bovis_BCG_str_Tokyo_172** |
| --- | --- | --- | --- | --- | --- | --- | --- |
| M_africanum_GM041182 | 10 | 10 | 10 | 6 | 7 | 7 | 7 |
| M_canettii_CIPT_140010059_3861 | 10 | 8 | 10 | 6 | 7 | 7 | 7 |
| M_canettii_CIPT_140010059 | 10 | 2 | 10 | 5 | 6 | 6 | 6 |
| M_canettii_CIPT_140060008 | 8 | 2 | 8 | 4 | 4 | 4 | 4 |
| M_canettii_CIPT_140070008 | 10 | 10 | 10 | 6 | 7 | 7 | 7 |
| M_canettii_CIPT_140070010 | 10 | 3 | 10 | 5 | 6 | 6 | 6 |
| M_canettii_CIPT_140070017 | 19 | 10 | 19 | 11 | 12 | 12 | 12 |
| Mtb_7199-99 | 20 | 10 | 20 | 11 | 12 | 12 | 12 |
| Mtb_CCDC5079 | 15 | 9 | 15 | 10 | 11 | 11 | 11 |
| Mtb_CCDC5180 | 16 | 10 | 16 | 12 | 13 | 13 | 13 |
| Mtb_CDC_1551 | 14 | 6 | 14 | 8 | 8 | 8 | 8 |
| Mtb_CTRI-2 | 20 | 10 | 20 | 11 | 12 | 12 | 12 |
| Mtb_F11 | 19 | 10 | 19 | 11 | 12 | 12 | 12 |
| Mtb_H37Rv | 20 | 10 | 20 | 11 | 12 | 12 | 12 |
| Mtb_H37Rv_broad | 11 | 3 | 11 | 5 | 6 | 6 | 6 |
| Mtb_KZN_1435 | 20 | 10 | 20 | 11 | 12 | 12 | 12 |
| Mtb_KZN_4207-3996 | 20 | 10 | 20 | 11 | 12 | 12 | 12 |
| Mtb_KZN_4207 | 10 | 3 | 10 | 5 | 6 | 6 | 6 |
| Mtb_KZN_605 | 20 | 10 | 20 | 11 | 12 | 12 | 12 |
| Mtb_KZN_R506 | 17 | 9 | 17 | 10 | 11 | 11 | 11 |
| Mtb_KZN_V2475 | 17 | 9 | 17 | 10 | 11 | 11 | 11 |
| Mtb_RGTB327 | 13 | 9 | 13 | 8 | 9 | 9 | 9 |
| Mtb_RGTB423 | 13 | 9 | 13 | 7 | 8 | 8 | 8 |
| Mtb_UT205 | 20 | 10 | 20 | 11 | 12 | 12 | 12 |

Table D: Searching the number of regions of variance of each tuberculoid strain from Non tuberculoid mycobacteria and vaccine strains. We have included *M africanum* GM041182 virulent strain as a representative of virulent strains.

|  |
| --- |

| **Tb strain** | **No. of RD proteins** | **M_africanum_GM041182** | **M_bovis_AF2122_97** | **M_bovis_BCG_str_Korea** | **M_bovis_BCG_str_Mexico** | **M_bovis_BCG_str_Pasteur_1173P2** | **M_bovis_BCG_str_Tokyo_172** |
| --- | --- | --- | --- | --- | --- | --- | --- |
| M_africanum_GM041182 | 23 | 23 | 14 | 12 | 13 | 13 | 13 |
| M_canettii_CIPT_140010059_3861 | 29 | 23 | 15 | 13 | 14 | 14 | 14 |
| M_canettii_CIPT_140010059 | 25 | 20 | 13 | 12 | 13 | 13 | 13 |
| M_canettii_CIPT_140060008 | 20 | 16 | 13 | 13 | 13 | 13 | 13 |
| M_canettii_CIPT_140070008 | 25 | 22 | 14 | 12 | 13 | 13 | 13 |
| M_canettii_CIPT_140070010 | 24 | 20 | 14 | 13 | 14 | 14 | 14 |
| M_canettii_CIPT_140070017 | 27 | 21 | 14 | 12 | 13 | 13 | 13 |
| Mtb_7199-99 | 33 | 23 | 17 | 13 | 14 | 14 | 14 |
| Mtb_CCDC5079 | 23 | 15 | 10 | 8 | 9 | 9 | 9 |
| Mtb_CCDC5180 | 26 | 18 | 12 | 10 | 11 | 11 | 11 |
| Mtb_CDC_1551 | 29 | 19 | 15 | 12 | 12 | 12 | 12 |
| Mtb_CTRI-2 | 30 | 22 | 15 | 13 | 14 | 14 | 14 |
| Mtb_F11 | 29 | 22 | 14 | 12 | 13 | 13 | 13 |
| Mtb_H37Rv | 33 | 23 | 17 | 13 | 14 | 14 | 14 |
| Mtb_H37Rv_broad | 30 | 22 | 16 | 13 | 14 | 14 | 14 |
| Mtb_KZN_1435 | 29 | 21 | 14 | 12 | 13 | 13 | 13 |
| Mtb_KZN_4207-3996 | 30 | 22 | 15 | 13 | 14 | 14 | 14 |
| Mtb_KZN_4207 | 28 | 20 | 14 | 13 | 14 | 14 | 14 |
| Mtb_KZN_605 | 30 | 22 | 15 | 13 | 14 | 14 | 14 |
| Mtb_KZN_R506 | 29 | 20 | 14 | 13 | 13 | 13 | 13 |
| Mtb_KZN_V2475 | 30 | 21 | 15 | 13 | 14 | 14 | 14 |
| Mtb_RGTB327 | 22 | 16 | 11 | 9 | 10 | 10 | 10 |
| Mtb_RGTB423 | 24 | 17 | 14 | 11 | 11 | 11 | 11 |
| Mtb_UT205 | 31 | 23 | 15 | 13 | 14 | 14 | 14 |

Table E: Selected vaccine candidate with their predicted immune response

| **Epitope** | **Non tuberculoid strain** | **No. of Tb strains** | **B cell epitope probability** | **MHC class II alleles** | **MHC class I alleles** | **HLA alleles** | **Th1 score** | **Th2 score** |
| --- | --- | --- | --- | --- | --- | --- | --- | --- |
| LRRPAPVKF | Mtb_H37Ra | 23 | 66.02 | 6 | 3 | 9 | 0.15 | 0.28 |
| YVPLTMGYY | Mtb_H37Ra | 23 | 66.09 | 8 | 6 | 14 | 0.18 | 0.18 |
| YLLADTFTV | M_kansasii_ATCC_12478, M_marinum, Mtb_H37Ra | 24 | 67.86 | 29 | 11 | 9 | 0.23 | 0.08 |
| VGAVTIVNL | M_kansasii_ATCC_12478, M_liflandiii_128FXT, M_marinum,Mtb_H37Ra | 24 | 63.78 | 2 | 10 | 12 | 0.03 | 0.4 |
| FAADVRANR | M_kansasii_ATCC_12478, Mtb_H37Ra | 23 | 64.53 | 2 | 5 | 9 | 0.12 | 0.45 |
| LRRATVIYG | M_indicus_prani_MTCC_9506, M_intracellulare_ATCC_13950, M_intracellulare_MOTT-02, M_intracellulare_MOTT-64, M_kansasii_ATCC_12478, Mtb_H37Ra, Mycobacterium_sp_MOTT36Y | 23 | 61.2 | 46 | 1 | 10 | 0.81 | 0.21 |
| WLTDRRREI | M_avium_K-10, M_kansasii_ATCC_12478, M_smegmatis_JS623, Mtb_H37Ra | 17 | 63.64 | 8 | 9 | 14 | 0.01 | 0.28 |
| FARAVRNAF |  | 2 | 61.23 | 3 | 8 | 10 | 0.23 | 0.4 |
| FEDRTVAML |  | 3 | 62.32 | 2 | 12 | 9 | 0.25 | 0.28 |
| FRHDRQNRL |  | 3 | 68.44 | 13 | 11 | 10 | 0.02 | 0.22 |
| MTPDPAVLV |  | 6 | 67.73 | 2 | 6 | 10 | 0.09 | 0.18 |
| VLRLRLLSK |  | 2 | 65.9 | 21 | 6 | 14 | 0.8 | 0.3 |
